# Multiple measures for self-identification improve matching donors with patients in unrelated hematopoietic stem cell transplant

**DOI:** 10.1038/s43856-024-00620-w

**Published:** 2024-10-03

**Authors:** Vincent Damotte, Chao Zhao, Chris Lin, Eric Williams, Yoram Louzoun, Abeer Madbouly, Rochelle Kotlarz, Marissa McDaniel, Paul J. Norman, Yong Wang, Martin Maiers, Jill A. Hollenbach

**Affiliations:** 1 UCSF Weill Institute for Neurosciences, Department of Neurology, University of California, San Francisco, CA USA; 2 Department of Computer Science, University of Washington, Seattle, WA USA; 3 Center for International Blood and Marrow Transplant Research, Minneapolis, MN USA; 4 National Marrow Donor Program / Be The Match, Minneapolis, MN USA; 5 Department of Mathematics, Bar-Ilan University, Ramat Gan, Israel; 6 Division of Personalized Medicine, and Department of Microbiology and Immunology, University of Colorado, Denver, Aurora, CO USA; 7 AncestryDNA, San Francisco, CA USA; 8 Department of Epidemiology and Biostatistics, University of California, San Francisco, CA USA

**Keywords:** Genetic testing, Transplant immunology

## Abstract

**Background:**

Questions persist around whether and how to use race or geographic ancestry in biomedical research and medicine, but these forms of self-identification serve as a critical tool to inform matching algorithms for human leukocyte antigen (*HLA*) of varying levels of resolution for unrelated hematopoietic stem cell transplant in large donor registries.

**Methods:**

Here, we examined multiple self-reported measures of race and ancestry from a survey of a cohort of over 100,000 U.S. volunteer bone marrow donors alongside their high-resolution *HLA* genotype data.

**Results:**

We find that these self-report measures are often non-overlapping, and that no single self-reported measure alone provides a better fit to *HLA* genetic ancestry than a combination including both race and geographic ancestry. We also found that patterns of reporting for race and ancestry appear to be influenced by participation in direct-to-consumer genetic ancestry testing.

**Conclusions:**

While these data are not used directly in matching for transplant, our results demonstrate that there is a place for the language of both race and geographic ancestry in the critical process of facilitating accurate prediction of *HLA* in the donor registry context.

## Plain language summary

Self-identification with respect to race and ancestry is an important component in the process of finding a matching unrelated bone marrow donor for a patient in large donor registries. Here, we considered whether terms specific to either race or the geographic ancestry of donors would be more useful in the matching process. We found that rather than using either of these terms alone, collecting responses for both race and geographic ancestry from potential donors is most likely to provide the information necessary to find a genetic match among millions of donors for a patient in need of a transplant.

## Abstract (web summary)

Damotte et al. examine the utility of multiple measures of race and ancestry self-identification in the context of matching HLA for potential unrelated bone marrow donors with patients. They show that combining both race and geographic ancestry provides a better fit to HLA than either measure alone.

## Introduction

While not used directly for matching donors and recipients, self-report data regarding race and ancestry are a critical part of the bioinformatic algorithm matching for human leukocyte antigen (*HLA*) of prospective donors with patients in need of hematopoietic stem cell transplant. Modern genomic methods may provide granular detail regarding ancestry, but genome-wide data is not collected routinely in bone marrow donor registries. Given the ongoing reliance on self-identification, how do we ensure that the methods that we employ for this critical task are best-suited toward inclusion of diverse populations? Beyond the transplant setting, these questions apply as well to the next generation of genomic, biomarker, behavioral research, clinical trials, and biobanks. Likewise, consideration of race continues to play a part in medical practice^1,2^. Historically, self-identification using race categories as defined by the United States Office of Management and Budget (OMB)^3^ has been standard; indeed, federally funded researchers are mandated to collect and report this information. Further complexity is added by inconsistent use of the term “ethnicity,” which is often used to describe a group sharing culture, language, or other features. However, many in the biomedical community have sought to focus rather on identification according to geographic ancestry^4–7^ It is argued that these measures better reflect human history and are more likely to represent biological differences compared to race, which is understood to be a social construct^8^. Meanwhile, there remains a need to incorporate some form of this information to expedite the matching process for patients in search of an unrelated bone marrow donor, where it is used to narrow the search space of possible donors by facilitating identification of the most likely high-resolution *HLA* haplotypes in the donor pool.

*HLA* data for potential volunteer unrelated donors stored in registries is of varying resolution; while *HLA* genotyping for donors recruited in the last several years is typically very high resolution (generally from sequence-based typing (SBT) methods) and complete with respect to loci genotyped, this varies for a substantial number of donors whose data were collected up to decades ago. Some data is incomplete with respect to the *HLA* loci typed (for example, often missing data for *HLA-C* or *HLA-DQB1*) and/or is low resolution (for example, was typed with serological methods or much lower resolution molecular methods). In order to perform efficient searches for a donor match for a given patient, these lower resolution *HLA* genotypes need to undergo algorithmic imputation to predict the most likely high-resolution *HLA* genotypes for a given donor. Among the inputs for this algorithm are known haplotypic associations in combination with known patterns of variation based on ancestry^9^—this is the primary use for self-identification data collected at the time of donor recruitment. Thus, while donors and patients are not matched according to race and ancestry, they are matched according to known or *predicted* high-resolution *HLA* genotype; the predicted genotypes having been informed, critically, by self-identified race and ancestry.

Although previous investigations have examined the relationship between single measures of self-identification and genetic ancestry^10–13^, here we expand on our earlier work considering self-identification for donors registered with the National Marrow Donor Program (NMDP)^14^ with an approach that differs from other studies in several important ways. We directly incorporate findings from the social sciences^15^ to perform a large-scale study comparing multiple measures of self-identification simultaneously with *HLA* genetic ancestry in the same cohort. Here, we specifically leverage genetic information for *HLA* to facilitate comparison between measures and understand whether some are more closely related to genetic ancestry in this region than others. We do so in a larger and more diverse sample of the U.S. adult population than previously examined, considering how both self-identified race and ancestry can be used to best describe human diversity, with a focus on the relevance for donor-patient matching algorithms^16^. In the National Marrow Donor Program Registry there are nearly 7 million volunteer bone marrow donors, the majority of which have missing or ambiguous typing at loci that are critical for matching. Population specific haplotype frequency data is used to make predictions but these predictions are only as good as the accuracy of the assignment of an individual to a population^17,18^. Finally, we consider the role that direct-to-consumer genetic testing may play in shifting patterns of self-identity, and the extent to which this may provide potential advantage in the registry context.

## Methods

We collected multiple self-reported measures of race and ancestry from a cohort of more than 100,000 U.S. adults who also provided genetic data for *HLA* as potential donors registered with the National Marrow Donor Program (NMDP). To ascertain genetic ancestry, we used the registry’s data for the human leukocyte antigen (*HLA*) complex on the short arm of chromosome 6, which is critical to matching in tissue transplant. The *HLA* loci exhibit extreme levels of variability and differentiation among human populations and the region is relatively well-maintained during gametogenesis, and thus can be used as ancestry informative markers^19–22^. Our survey of potential NMDP donors, conducted for this study in spring 2015, included questions about racial self-identification and multiple (geographic) ancestry items. All participants provided informed consent (available in Supplementary files) and this study was approved by the Institutional Review Board at the University of California San Francisco (study #14-13977).

### Survey questions

For self-reported ancestry, we included three measurement approaches: (1) personal ancestry (PA), a check-all-that-apply option using a series of geographic categories; (2) personal ancestry salience (PAS), a measure that asked people to “weight” their ancestry self-reports on a 100-point scale; and 3) family ancestry (FA), check-all-that-apply ancestry questions about specific biological relatives, such as grandparents. In order to fully exploit the FA responses, we also computed a summary family fractional ancestry (FFA) value from the family responses based on the number of ancestry selections per parent or grandparent (Supplementary Methods). In addition to asking respondents to describe themselves using official racial categories (RC), we also asked that they tell us how they think other Americans would classify them using the same categories, which we term “reflected race” (RR)^23^; we were interested in this measure as a proxy for race coding that might be contributed by a third party, such as a clinical provider. The complete survey is provided in the Supplementary Material.

### Assignment of HLA haplotype ancestry

To understand how these measures of self-reported race and ancestry relate to genetic ancestry, we employed a Bayesian classifier to assign the most probable geographic origin for subjects’ *HLA* haplotypes (Supplementary Table S1). Our previous work had shown that population-level *HLA* haplotype ancestry assignments using this method are equivalent to ancestry proportions derived from a well-characterized panel of ancestry informative markers^14^. To further validate the classifier, we examined prediction of the *HLA*-based ancestry classifications from ancestry proportions derived from over 700,000 single nucleotide polymorphism (SNP) markers for an independent dataset of 1983 individuals, with cross-validation revealing accuracy approaching 85% (Supplementary Fig. S1).

### Data analysis

We tested the fit of all self-reported race and ancestry responses alone and in specific combinations as predictors of genetic (*HLA* haplotype) ancestry in a multinomial logistic regression model, including covariates for age, sex, and educational attainment (Detailed in Supplementary Methods). Our survey methodology included randomly switching the order in which the race vs. ancestry sections were presented, which yielded some variation in the number of responses for each section, and thus we adjusted for this feature. Likewise, we adjusted for the email outreach recruiting participants, the specific language of which varied (Supplementary Fig. S2). To test for genetic differences between groups of respondents, we calculated Edward’s genetic distance and tested for significance using a permutation procedure (Detailed in Supplementary Methods).

### Statistics and reproducibility

All data analysis was performed in the R environment for statistical computing. All analysis is reproducible using the code linked below in “Code availability^24^.”

### Reporting summary

Further information on research design is available in the Nature Portfolio Reporting Summary linked to this article.

## Results

### The relationship between measures of self-reported race and ancestry is complex and non-redundant

**Fig. 1:**
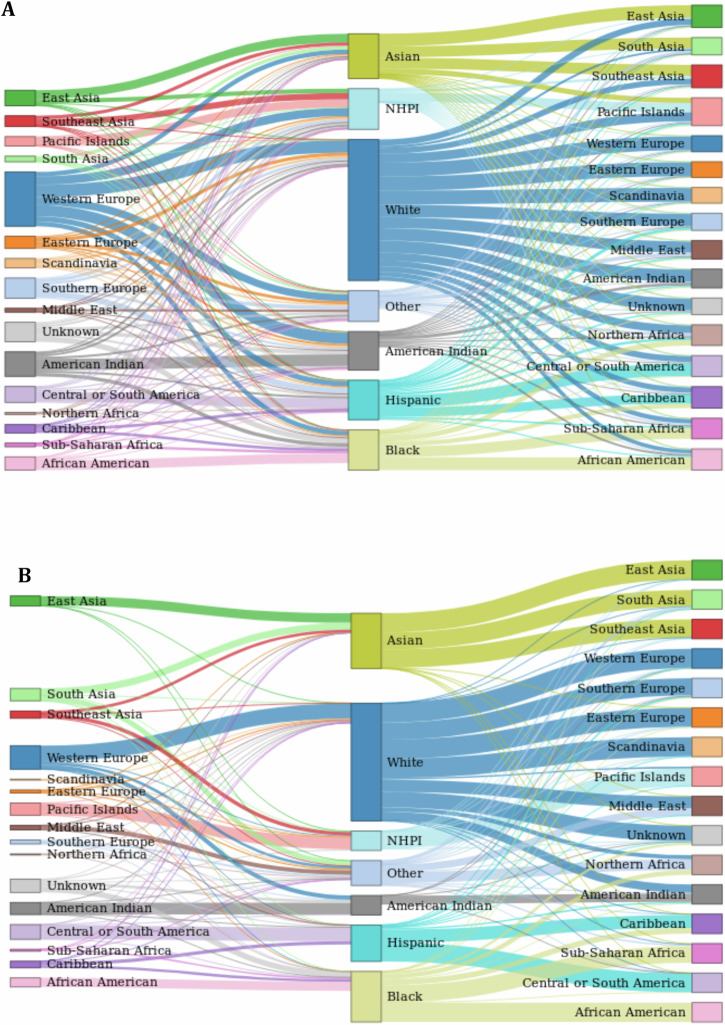
Sankey diagram of connection between racial categories and geographic ancestries selected by respondents. **A** Considering all respondents. **B** Only respondents who selected a single race category and a single geographic ancestry were considered.

Respondent demographics detailed by sex and age with respect to place of birth and response to RC are shown in Tables 1 and 2, respectively. Despite often being treated interchangeably, we found that measures of self-reported race and ancestry are often non-overlapping, even when administered simultaneously in the same cohort. On the surface, responses for RC and PA might seem to provide redundant information, with many respondents identifying as White and also reporting PA from Western Europe, for example. However, cross-tabulating the measures with one another showed they are not as interchangeable as they might appear at first glance. When comparing racial self-identification and PA, every possible PA was connected to every possible RC in our sample (Fig. 1A), yielding a total of 3,582 different RC/PA combinations (Supplementary Table S2). Nearly 60% of the sample self-reported two or more PA responses, and close to 12% provided two or more RC responses. Even when we restrict to individuals who selected a single PA and single RC response to describe themselves (39% of our sample), much of the complexity between ancestry and race reporting remains (Fig. 1B).

**Table 1:** Survey respondent demographics (gender and age groups) separated by place of birth

	Total	Female	Male	[18–24]	[25–34]	[35–44]	[45–54]	[55–64]	[65+]
All respondents	103348	82226	21121	13576	35487	27804	18105	8313	9
*Respondent US born*									
Yes	95770	80.1%	19.9%	13.3%	34.5%	26.6%	17.4%	8.2%	0%
No	7487	72.7%	27.3%	11.4%	32.8%	31.1%	18.5%	6.1%	0%
*Parents US born*									
Neither	11107	73.8%	26.2%	16.7%	35.6%	27.4%	15.4%	4.9%	0%
One	8190	80.7%	19.3%	17.2%	35.9%	26.2%	14.5%	6.2%	0%
Both	84012	80.2%	19.8%	12.3%	34.0%	26.9%	18.1%	8.6%	0%
*Grandparents US born*									
None	15319	74.7%	25.3%	14.8%	31.7%	25.6%	18.7%	9.1%	0%
One	3031	79.8%	20.2%	12.9%	27.6%	25.1%	22.3%	12.1%	0%
Two	13639	80.6%	19.4%	13.6%	31.7%	25.1%	19.5%	10.1%	0%
Three	9939	81.8%	18.2%	12.9%	34.8%	26.1%	17.7%	8.6%	0%
Four	61365	80.2%	19.8%	12.7%	35.9%	27.9%	16.5%	7.0%	0%

Gender and age group were missing for 1 and 63 individuals, respectively. Place of birth was missing for 91 individuals, parents place of birth was missing for 39 individuals. Grand-parents place of birth was missing for 235 individuals.

**Table 2:** Survey respondents demographics (gender and age groups) separated by race

	Total	Female	Male	[18–24]	[25–34]	[35–44]	[45–54]	[55–64]	[65+]
All respondents	103348	82226	21121	13576	35487	27804	18105	8313	9
American Indian	279	80.6%	19.4%	10.4%	22.9%	35.8%	24.0%	6.8%	0%
Asian	3461	67.6%	32.4%	18.2%	42.1%	25.1%	11.2%	3.4%	0%
Black	3044	84.3%	15.7%	14.1%	30.7%	29.1%	18.4%	7.7%	0%
Hispanic	4889	80.6%	19.4%	21.5%	34.9%	27.5%	12.4%	3.7%	0%
Native Hawaiian or Pacific Islander	129	76.7%	23.3%	10.1%	34.9%	36.4%	12.4%	6.2%	0%
White	78489	79.8%	20.2%	11.5%	33.4%	27.0%	18.9%	9.1%	0%
Other	1146	66.8%	33.2%	11.2%	34.9%	28.4%	17.0%	8.5%	0%
Multi-race	11903	81.4%	18.6%	18.9%	39.2%	25.4%	11.9%	4.5%	0%

Gender and age group were missing for 1 and 63 individuals, respectively.

### Combining self-identification responses for race and ancestry provides the best fit to HLA variation

**Fig. 2:**
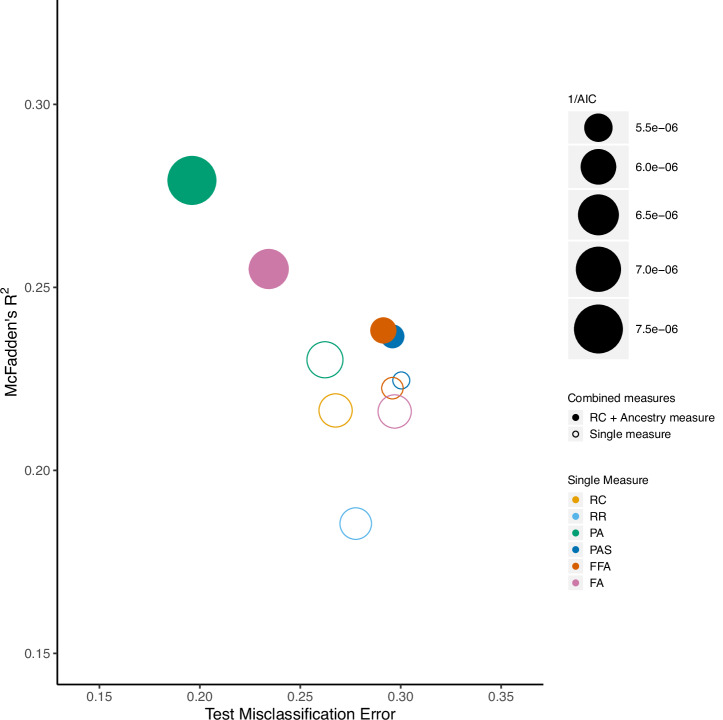
Assessment of different races and/or ancestries models. These models represent the observed fits of different models as predictors of genetic (*HLA* haplotype) ancestry (see Materials and Methods). Shown on the x-axis is the test misclassification error (rate of incorrect model prediction) and values for McFadden’s R2 are shown on the y-axis, which corresponds to goodness of fit. The predictors shown are as follows: RC race category; PR personal race; RR reflected race; PA personal ancestry; PAS personal ancestry salience; FFA fractional family ancestry; FA family ancestry.

We found that no single self-reported measure of race or ancestry alone provides a better fit to *HLA* genetic ancestry classification than combined measures (Fig. 2). When examining single measures, PA provided the best model fit, lending support to the notion that geographic ancestry serves as a better proxy for genetic ancestry than self-identified race. However, RC provided a better fit than any of our other single measures, including FA, while RR fit very poorly, with the lowest *R*^2^ of any measure. Our quantitative measures, PAS and FFA, were highly correlated (Supplementary Table S3), but had the highest misclassification rates of any single measure we examined, diminishing the overall model fit. Although PA provided better fit than the RC response alone, fit to *HLA* genetic ancestry was significantly improved by incorporating the RC response with any of the ancestry measures, with the most significant improvements noted for combinations including PA and FA. Strikingly, the best-fitting model predicting genetic ancestry classification included a combination of RC self-identification and PA. This combined measure showed marked improvement in model fit compared to the PA single measure (*p* < 0.001).

Specific examples from our data illustrate why combining race and ancestry responses serves to better represent genetic variation than single measures of self-identification. For instance, complexity in reporting American Indian race and ancestry is well documented in demographic studies^25,26^. American Indian PA is reported frequently in our sample (15% of individuals), and is most often seen in combination with Western European PA (N = 5709). Despite the fact that “American Indian” is also provided as an option for the RC response, many individuals reporting this PA combination report only the White RC. We computed the *HLA* genetic distance between individuals reporting the specific combination of Western Europe and American Indian PA with only White RC (80%) and those who reported the same PA (Western Europe and American Indian) with White RC plus American Indian RC (17%) or only American Indian RC (1.6%); using a permutation procedure, we found that the White-only RC and White RC plus American Indian RC groups are not significantly divergent (*p* = 0.15). However, the American Indian-only RC group is significantly divergent from the White-only RC group (*p* < 0.001) and from the White RC plus American Indian RC group (*p* = 0.03), showing the added value of combining race and ancestry responses in more accurately imputing high-resolution *HLA* for transplant matching.

**Fig. 3:**
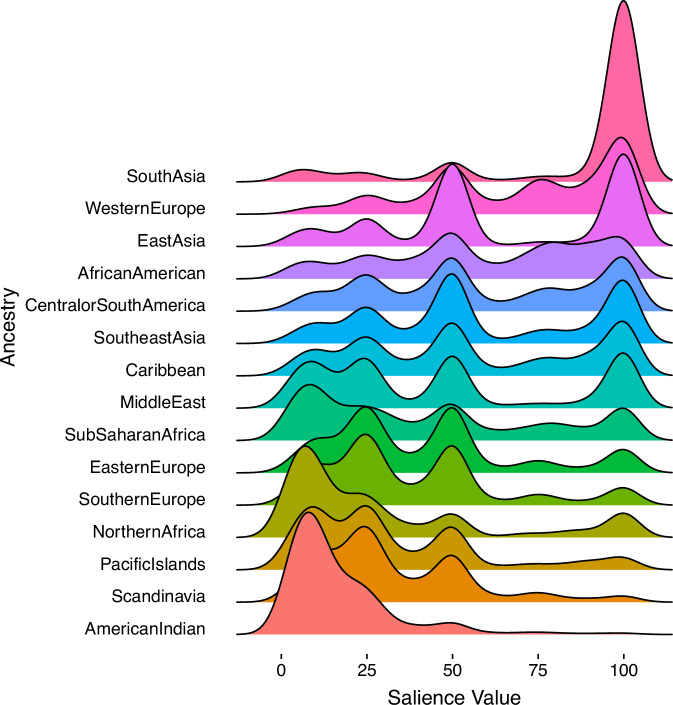
Density plots of personal ancestry salience (PAS) values given by individuals who selected specific geographic ancestry. The x-axis represents salience values (range 0-100) provided by participants for specific ancestries.

Whereas incorporation of salience values (PAS) did not improve the overall fit of our models, they do provide important insights into the underlying dynamics in ancestry identification. Although frequently reported, American Indian PA yields the lowest mean PAS value (16.8) of any PA response (Fig. 3). Even among individuals who report American Indian FA for all four of their biological grandparents, their mean American Indian PAS value is only 49; in comparison, individuals who report four South Asian grandparents FA report South Asian mean PAS of 99 (*p* < 0.001). These results may also explain why PA provided better overall model fit to *HLA* variation than FA. Notably, individuals who identify with American Indian RC report significantly higher American Indian PAS than those who did not (mean 26 vs. 14; *p* < 0.001).

Likewise, we observed complexity comparing racial self-identification as Black with sub-Saharan African PA, furthering support for combining measures of self-identified race and ancestry in the matching algorithms designed to improve resolution for *HLA* variation. Although tracing ancestry to the original peoples of sub-Saharan Africa is the official definition of the “Black or African American” racial category in the U.S.^3^, we offered both “Sub-Saharan Africa” and “African American” categories among our ancestry responses. Among respondents who identified RC as Black alone (*N* = 3038), 67% reported African American PA, compared to 17% who reported Sub-Saharan African PA. We analyzed the *HLA* genetic distance between individuals who identified as Black RC alone and who reported African American ancestry only and those who reported Sub-Saharan African ancestry only and found significant divergence (*p* < 0.001). One explanation for these observations may be found in respondents’ nativity: among respondents who identified as Black RC alone, respondents who reported sub-Saharan African ancestry were significantly less likely to have been born in the U.S. than those who did not report this ancestry (84% and 93% respectively; *p* < 0.001). Foreign-born Black RC respondents who reported sub-Saharan African PA also reported a mean sub-Saharan African PAS value of 82, compared to 45 for their U.S.-born counterparts who selected the same RC and PA responses (*p* < 0.001).

### Participation in direct-to-consumer ancestry testing changes patterns of self-identification

Finally, we found that self-identification reporting patterns may be transformed by participation in direct-to-consumer genetic ancestry testing (GAT). Approximately 5% of our respondents reported having taken a GAT^27^. Overall, these individuals gave more responses for ancestry (mean responses 2.3 vs 1.9; *p* < 0.001) as well as distinctive combinations of race and ancestry reporting compared to those who did not use GAT. Among respondents who identified as Black RC alone, 62% reported sub-Saharan African PA if they had taken a GAT compared to 14% who have never taken a GAT (*p* < 0.001). In contrast, these groups reported African American PA nearly equivalently at 70% and 66%, respectively. In contrast to the larger sample, genetic distance measures were non-significant between Black RC individuals who did or did not report sub-Saharan African PA. Likewise, among GAT participants, 96% of Black respondents reporting sub-Saharan ancestry also reported being U.S. born. In addition to sub-Saharan African PA, a number of other PA responses were also found to differ in frequency according to whether respondents had used GAT. For example, among GAT takers, American Indian PA was reported less often by individuals identifying as White RC (*p*_corr _= 0.004), but more often by individuals identifying as Hispanic RC (*p*_corr _< 0.001) compared to those who did not use GAT. Thus, in contrast to individuals who did not participate in GAT, here the race response did not improve model fit and racial identification appears not relevant with respect to *HLA* genetic ancestry.

## Discussion

Taken together, our results demonstrate that there is a place for the language of both race and (geographic) ancestry in the specific context of matching for *HLA* in donor registries. Although not used for matching itself, given the high level of ambiguity in *HLA* genotyping in donor registries and the critical role that self-identified race and ancestry play in bioinformatic predicting high-resolution, unambiguous *HLA* genotypes, more accurate self-identification of race and ancestry translates directly to more accurate matching and improved patient outcomes. A limitation of this study is the lack of genome-wide data for comparison to results for the *HLA* data. Given the objective here to consider prediction of high-resolution *HLA* genotypes, we acknowledge that the results might not be applicable to questions considering the relationship between genetics and self-identification outside of the registry setting. Likewise, these results are specific to the context of a U.S. donor registry and may not be applicable to other populations, which may be significantly more homogenous or have very different histories of immigration.

Consideration of multiple measures here has revealed the underlying complexity in self-identification, with substantial variance between ancestries. For example, we show that while a substantial number of respondents claim American Indian *ancestry*, many acknowledge its relatively low salience; for those who do not simultaneously identify as American Indian in the context of *race*, we did not observe significant deviation in terms of *HLA* genetics from those who did not identify with this ancestry. In contrast, individuals who do select American Indian in the context of race typically gave higher salience values to that ancestry and were genetically distinct from those who did not. Thus, in this case racial self-identification appears to signal both personal and biological relevance. Our results also illustrate one of the pitfalls of using a check-all-that-apply format for reporting geographic origins as the sole self-identification measure in the registry setting.

Examination of *HLA* genetic differentiation among respondents who identified as Black in the context of race, but have variably selected between African American and Sub-Saharan African in the context of geographic ancestry, underscores conversely the pitfalls in using race as the only measure of self-identification. Here, although shared racial identification suggests a shared social experience of “blackness,” which likely has implications for health^28,29^, a registry that groups donors solely by racial self-identification might miss the *HLA* genetic variation among individuals and their differing immigration histories, which could be important for matching as well as understanding match disparities. For some other ancestries, racial self-identification has even more limitation. A high proportion of individuals claiming only Middle Eastern or North African ancestry do not identify with any of the standard OMB RC’s, and rather select Other. Likewise, South Asian ancestry is generally split between the Other category and Asian RC (Fig. 1).

These results underscore the notion that race and ancestry are describing distinct aspects of self-identification, which partially – but far from completely – overlap. Moreover, these patterns vary by population, emphasizing the need to embrace multiple measures in order to offer appropriate options to diverse cohorts. Accordingly, our results show that while providing important information, self-reported geographic ancestry alone is not as good a proxy for genetic variation in the context of *HLA* as when coupled with racial self-identification; there is also ample research that shows self-reported race has a role to play in studies of health disparities, and thus it might be important for the continued collection of this information by registries to continue to track and ameliorate longstanding inequalities in match rates across racial groups. Our results for individuals participating in GAT suggest that as genealogical tools and technologies increase in popularity and accessibility, individuals may move toward means of self-identification that are more geographically, and less racially, based; this may present an important opportunity for donor registries going forward as increasing numbers of engaged donors employ GAT.

In conclusion, this work demonstrates that we stand to improve current matching algorithms by recognizing the differences between measures of race and ancestry, and leveraging the instances of empirical convergence and divergence presented here to better reflect modes of identification that resonate with donors.

## Supplementary information

Supplementary Information

Description of Additional Supplementary Files

Supplementary Data Table 1

Reporting Summary

## Data Availability

The full raw data that support the findings of this study are not openly available due to reasons of sensitivity and are available from the corresponding author upon reasonable request. Data are located in controlled access data storage at the University of California San Francisco. Processed data underlying all figures and tables (source data) are given in Supplementary Data Table 1.

## Code availability

Analytical codes for this study are available at https://github.com/Hollenbach-lab/AQP_Paper1_PublicRelease, 10.5281/zenodo.13685515^24^.
