# Supplementary material for: Multiple measures for self-identification improve matching donors with patients in unrelated hematopoietic stem cell transplant
Source: Commun Med (Lond). 2024 Oct 3;4:189. doi: 10.1038/s43856-024-00620-w (PMC11449941; doi:10.1038/s43856-024-00620-w)
Supplement: Supplementary file 1 — Supplementary Information [file 43856_2024_620_MOESM1_ESM.pdf]

Multiple Measures Reveal the Value of Both Race And Geographic Ancestry For Self-Identification in Matching Donors with Patients in Unrelated Hematopoietic Stem Cell Transplant

Authors: Vincent Damotte[1], Chao Zhao[1], Chris Lin[2], Eric Williams[3,4], Yoram Louzoun[5], Abeer Madbouly[3,4], Rochelle Kotlarz[3,4], Marissa McDaniel[4], Paul J. Norman[6], Yong Wang[7], Martin Maiers[3,4+], Jill A. Hollenbach[1,8+*]

Affiliations:

[1]UCSF Weill Institute for Neurosciences, Department of Neurology, University of California, San Francisco, CA, USA

[2]Department of Statistics, Stanford University, Stanford, CA, USA

[3]Center for International Blood and Marrow Transplant Research, Minneapolis, MN, USA

[4]National Marrow Donor Program / Be The Match, Minneapolis, MN, USA

[5]Department of Mathematics, Bar-Ilan University, Ramat Gan, Israel

[6]Division of Personalized Medicine, and Department of Microbiology and Immunology, University of Colorado, Denver, Aurora, CO, USA

[7]AncestryDNA, San Francisco, CA, USA

[8]Department of Epidemiology and Biostatistics, University of California, San Francisco, CA, USA

+Authors contributed equally to this work.

*Correspondence to:

Jill A. Hollenbach, PhD, MPH

jill.hollenbach@ucsf.edu

Supplemental Information

Methods. Supplementary methods
Table S1. Number of races (rows) and ancestries (columns) selected
Figure S1. Cross-validation of ancestry proportions
Figure S2. The two different versions of email outreach for recruiting participants
Table S2. Correlation of fractional family ancestry (FFA) with personal ancestry salience (PAS)
Survey. Survey sent to participants
Supplementary References

Methods: Supplementary methods

Analytical codes for this study are available at: https://github.com/Hollenbach-lab/AQP_Paper1_PublicRelease
Request for data access must be sent to the corresponding author.

A. Data cleaning

- Race data: Individuals with a non-available observation in at least one variable were removed.
- Personal Ancestry data: Individuals with a non-available observation in at least one variable were removed.
- Reflected Race data: Individuals with a non-available observation in at least one variable were removed.
- Personal Ancestry Salience data: Individuals with a non-available observation in at least one variable were removed. Only individuals with a sum of all salience different from zero were kept.
- Family Ancestry data: Family data were divided into two types of data: Individuals who selected ancestry data for their four grandparents and, when ancestry data were missing for at least one grandparent, we used the ancestry information selected for their two parents. Individuals with a non-available observation in at least one variable for the ancestry for their four grandparents <u>or</u> in at least one variable for the ancestry of their two parents were removed.
  First, per ancestry and per grandparent, the ancestry binary variables were transformed into percentages, by multiplying the binary outcome by 0.25. Then the same ancestry variables of each grandparent were summed up. Second, for individuals without ancestries for their four grandparents and with ancestries for at least one of their parents, ancestry variables were computed based on their parents ancestry information. Per ancestry and per parent, the ancestry binary variables were transformed into percentages, by multiplying the binary outcome by 0.50. If one of the parents did not have any ancestry information, we considered a percentage of 0.5 for the *Unknown* ancestry and 0 for the other choices for this parent. Next, the same ancestry variables of each parent were summed and the parents and grandparents ancestry percentages were bound to create the fractional family ancestry (FFA) data.

B. Descriptive analyses

The Sankey diagrams were made with the function *sankeyNetwork* in the R package networkD3[1]. All the other plots were made with the R package ggplot2[2]. Only ancestries weighted strictly above 0% were included for the ridgeline PAS analysis. Comparisons of PAS values between two groups were performed using a t-test. Comparisons of group size were performed using a Fisher test. Pearson's correlation coefficients were computed, and the correlation coefficients were tested for significance using Fisher's Z transformation.

C. Bayesian classifier to assign the most probable geographic origin for subjects' HLA haplotypes

The subjects were typed using a variety of PCR based DNA sequencing method targeting of HLA-A, -B, -C, -DRB1 and -DQB1[3]. Subject were typed at 3 different laboratories from 2008 – 2014 during a period where the laboratory achieved an average unphased typing resolution score[4] of at least 0.75. The resulting genotypes have no phase information between loci but only modest amounts of ambiguity in terms of allele assignments within a locus. The genotypes were analyzed using an updated "multi-race" version of an imputation algorithm described previously[5] that generated: a list of all possible pairs of 5-locus phased, allele-level haplotypes with corresponding haplotype frequency and population origin for each, including cases where the two populations can either be the same or different.
The population frequencies used for this study were an updated version of those published previously[6]. 18 populations sets were included in the imputation process which are the 18 categories listed in the table below.

Race code	Detailed race/ethnic description
**AAFA**	African American
**AFB**	African
**AINDI**	South Asian Indian
**AMIND**	North American Indian
**CARB**	Caribbean black
**CARHIS**	Caribbean hispanic
**CARIBI**	Caribbean Indian
**EURCAU**	European caucasian
**FILII**	Filipino
**HAWI**	Hawaiian or other Pacific Islander
**JAPI**	Japanese
**KORI**	Korean
**MENAFC**	Middle Eastern or N. Coast of Africa
**MSWHIS**	Mexican or Chicano
**NCHI**	Chinese
**SCAHIS**	Hispanic – South or Central American
**SCSEAI**	Southeast Asian
**VIET**	Vietnamese

Table S1. Population sets included in the imputation process

D. Estimate of haplotype ancestry combination based on estimated single ancestry haplotype frequency distributions.

Given the possible genotypes of an individual *i* and a set of haplotype frequencies for each sub population – *k*, and haplotype h_j : $f_k(h_j)$, we first estimate the probability of each haplotype pair in each population pair $f_k(h_{j1}), f_l(h_{j2})$, where h_{j1}, h_{j2} are a pair of haplotypes consistent with the genotype of individual *i.* We then estimate the total probability that individual *i* haplotypes originated from the population pair k,l - $p(k,l) = \sum_{j1,j2} f_k(h_{j1}), f_l(h_{j2})$, where the sum is only on haplotypes consistent with the genotype of individual *i.* The race combination maximizing $p(k,l)$ is defined as the race combination of individual *i*. Given the race combination, we estimate the most probable haplotype on race k, as $h_1 = ArgMax(f_k(h_{j1}))$. The second haplotype is then defined as $h_2 = \underset{h_{j2}}{ArgMax}(f_l(h_{j2}) * f_k(h_1))$.

E. Estimation of ancestry proportions in validation sample

Admixture proportions were estimated for all 2,005 samples using ADMIXTURE[7]. Each sample was analyzed separately in ADMIXTURE's "supervised" mode (--supervised) by comparing its genotypes with a pre-curated population reference panel consisting of 3,000 labeled reference samples from 26 global populations. The default block relaxation optimization method was utilized and the convergence criterion was set to a change in log-likelihood between two iterations falling below 0.01 (-C 0.01). In addition, standard errors were estimated for the admixture proportions by running ADMIXTURE with 40 bootstrap replicates (-B 40).

Population reference panel candidates were selected from the publicly accessible Human Genome Diversity Project[8,9], an internal proprietary AncestryDNA reference collection, and a reference collection corresponding to AncestryDNA customers who explicitly provided prior consent to participate in research and have all family lineages tracing back to the same geographic region. All the candidates were analyzed through a quality control

pipeline to remove samples with lower genotype call rate, samples genetically related to each other, and samples who appear as outliers from their purported population of origin based on Principal Component Analysis (PCA). Note, PCA analyses were performed using a subset of independent SNPs following linkage disequilibrium (LD) filtering. Using PLINK[10], one of each pair of high LD SNPs in a 50-SNP sliding window were removed. The final ethnicity reference panel contains 3,000 samples representing 26 distinct global populations. For more details, please see AncestryDNA's methods white paper[11].

F. Bayes classifier cross-validation

We used multinomial logistic regression to predict population assignments derived from the Bayes classifier from ancestry proportions derived from SNP data (as described above) in our validation cohort. The models were assessed by 10-fold cross-validation. The sample (N = 1981) was randomly partitioned into 10 approximately equal size subsamples. A single subsample is retained as the validation data for testing the model, and the remaining 9 subsamples are used as training data. The predictive accuracy was measured by root-mean-square error (RMSE) on both training data and test data. Our results indicate a testing misclassification error of only 16% (Additional File 3), indicating very good agreement between the two methods of ancestry determination.

G. Haplotypes clustering-based modeling and multinomial logistic regression

1. *Races and Ancestries data preparation*

General cleaning previously performed was used to prepare the following survey data.

- Race (RC): No additional cleaning was done. Binary data for each variable, 7 variables.
- Personal Ancestry (PA): Individuals who only chose *Unknown* ancestry were removed. Binary data for each variable, 16 variables.
- Race + Personal Ancestry (RC/PA): The Race and Personal Ancestry information created above were combined. Binary data for each variable, 23 variables.
- Reflected Race (RR): The Reflected Race variable with 7 levels was converted into 7 variables: American Indian or Alaska Native, Asian, Black or African American, Hispanic or Latino, Native Hawaiian or other Pacific Islander, White and Other. Binary data for each variable, 7 variables.
- Personal Ancestry Salience (PAS): The Personal Ancestry Salience was used. No additional cleaning was done. Percentages data for each variable, 16 variables.
- Race + Personal Ancestry Salience (RC/PAS): The Race and Personal Ancestry Salience information created above were combined. Binary data for Personal Race variables and Percentages data for each Personal Ancestry Salience variable, 23 variables.
- Fractional Family Ancestry (FFA): Percentages (0 to 100%) data for each variable as prepared previously (see above methods for data cleaning), 16 variables.
- Race + Fractional Family Ancestry (RC/FFA): The Race and Fractional Family Ancestry information created above were combined. Binary data for Races variables, Percentages (0 to 100%) data for Fractional Family Ancestry variables, 23 variables.
- Family Ancestry (FA): Above Fractional Familial Ancestry percentages (FFA) were replaced by 1 or 0, whether they were strictly higher than 0% or not. Binary data for each variable, 16 variables.
- Race + Family Ancestry (PR/FA): Race and Family Ancestry information created above were combined. Binary data for each variable, 23 variables.

Commons IDs: Only individuals present in each of these models were kept for subsequent analyses.

2. *K-means Clustering Algorithm*

Among 90,731 participants, there were 171 different unique haplotypes combinations based on 18 haplotypes. The unsupervised k-means clustering algorithm was performed to regroup and reduce the haplotypes levels for classification. The number of clusters was validated by the elbow method which run k-means clustering on the dataset for a range of values of k from 1 to 50, and the sum of squared errors (SSE) were calculated for each value of k. A smaller k value (k = 18) with a lower SSE was determined from the total within-cluster sum of squares plot (wss-plot) and was used to replace the 171 different unique genetic outcomes. The 18 initial "means" were randomly generated within the data domains. Each data point was assigned to its closest cluster center according to the Euclidean distance function. The new centroid or mean of all objects in each cluster were calculated. Each data point was reassigned to the new centroid. The steps were repeated until convergence has been reached.

3. Multinomial Logistic Regression

Multinomial logistic regression (MLR) was used to investigate the relationship between the population assignments based on HLA with race and ancestry information among 90,731 participants. MLR is designed to deal with cases of dependent variables with multiple classes. One major advantage of MLR is it is robust to violations of assumptions of multivariate normality as is the case where there are some zero/one variables or where distributions are highly skewed or heavy-tailed. The strength of the MLR relationship between dependent variable and independent variables was estimated by correlation measure (pseudo R squares measures, such as McFadden's R2). Values of McFadden's R^2 between 0.2 and 0.4 are considered highly satisfactory of goodness of fit. To assess the strength of MLR relationship, the evaluation of the usefulness for logistic models was also considered. We assessed misclassification errors on both training set (81,657 participants) and test set (9,074 participants), which compared the predicted groups to the actual groups. MLR was performed using the R package *nnet*[12].

H. Methods for Edward's Genetic Distances analyses

2,118 SNPs genotypes from the HLA region were available for 102,982 surveyed individuals. 443 SNPs were polymorphic and kept for subsequent analysis.

The adegenet R package[13,14] was used to compute Edward's genetic distances. Only populations with more than 50 individuals were kept for analyses. 999 permutations were performed to obtain one-tailed p-values testing the genetic distances differences between two populations. A p-value below 0.05 signifies that no more than 49/999 permutations led to distances higher than the one observed.

Figure S1. Cross-validation of ancestry proportions.

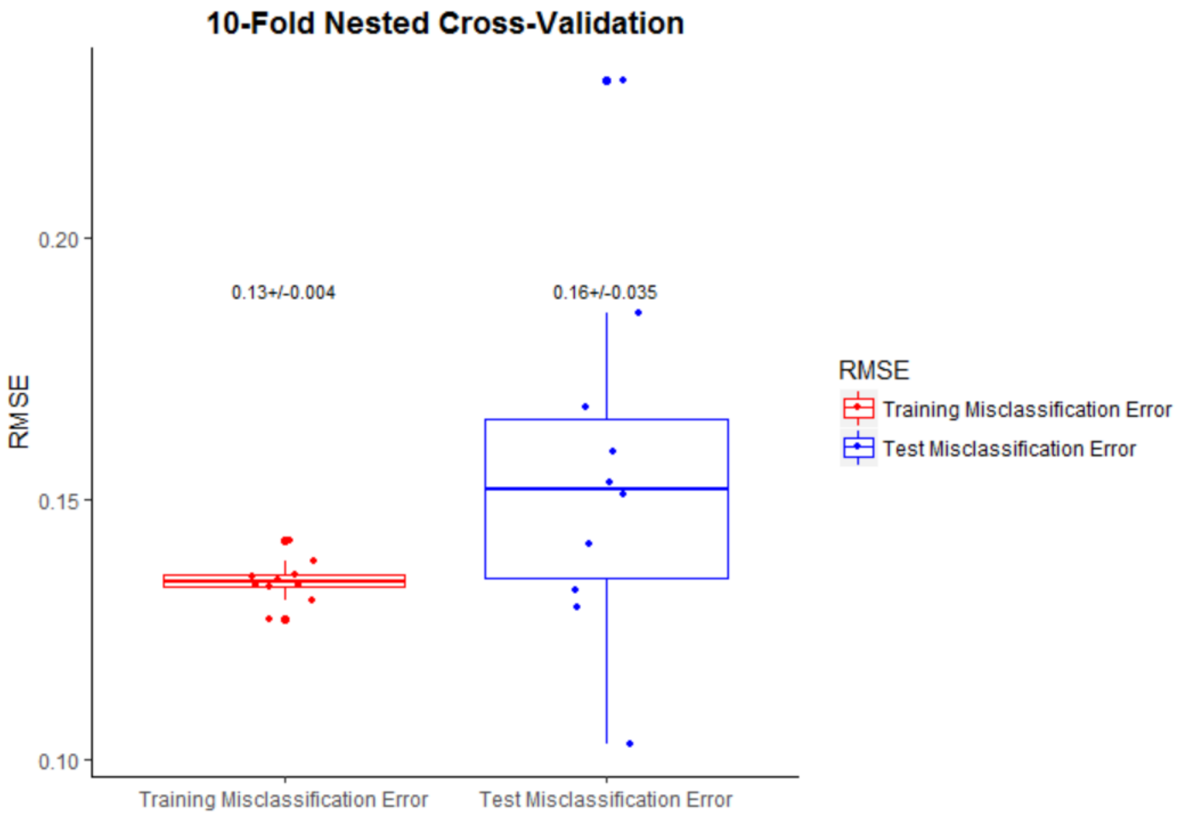

Figure S2. The two different versions of email outreach for recruiting participants.

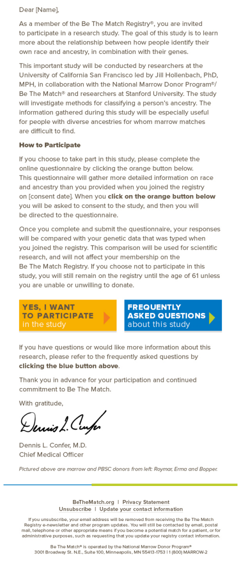

Dear [Name],

As a member of the Be The Match Registry®, you are invited to participate in a research study. The goal of this study is to learn more about the relationship between how people identify their own race and ancestry, in combination with their genes.

This important study will be conducted by researchers at the University of California San Francisco led by Jill Hollenbach, PhD, MPH, in collaboration with the National Marrow Donor Program®/Be The Match® and researchers at Stanford University. The study will investigate methods for classifying a person's ancestry. The information gathered during this study will be especially useful for people with diverse ancestries for whom marrow matches are difficult to find.

How to Participate

If you choose to take part in this study, please complete the online questionnaire by clicking the orange button below. This questionnaire will gather more detailed information on race and ancestry than you provided when you joined the registry on [consent date]. When you **click on the orange button below** you will be asked to consent to the study, and then you will be directed to the questionnaire.

Once you complete and submit the questionnaire, your responses will be compared with your genetic data that was typed when you joined the registry. This comparison will be used for scientific research, and will not affect your membership on the Be The Match Registry. If you choose not to participate in this study, you will still remain on the registry until the age of 61 unless you are unable or unwilling to donate.

YES, I WANT TO PARTICIPATE in the study

FREQUENTLY ASKED QUESTIONS about this study

If you have questions or would like more information about this research, please refer to the frequently asked questions by **clicking the blue button above**.

Thank you in advance for your participation and continued commitment to Be The Match.

With gratitude,

Dennis L. Confer, M.D.
Chief Medical Officer

Pictured above are marrow and PBSC donors from left: Roymar, Erma and Bopper.

BeTheMatch.org | **Privacy Statement**
Unsubscribe | **Update your contact information**

If you unsubscribe, your email address will be removed from receiving the Be The Match Registry e-newsletter and other program updates. You will still be contacted by email, postal mail, telephone or other appropriate means if you become a potential match for a patient, or for administrative purposes, such as requesting that you update your registry contact information.

Be The Match® is operated by the National Marrow Donor Program®
3001 Broadway St. N.E., Suite 100, Minneapolis, MN 55413-1753 | 1 (800) MARROW-2

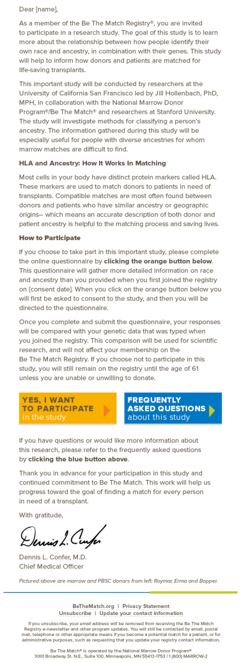

Dear [name],

As a member of the Be The Match Registry®, you are invited to participate in a research study. The goal of this study is to learn more about the relationship between how people identify their own race and ancestry, in combination with their genes. This study will help to inform how donors and patients are matched for life-saving transplants.

This important study will be conducted by researchers at the University of California San Francisco led by Jill Hollenbach, PhD, MPH, in collaboration with the National Marrow Donor Program®/Be The Match® and researchers at Stanford University. The study will investigate methods for classifying a person's ancestry. The information gathered during this study will be especially useful for people with diverse ancestries for whom marrow matches are difficult to find.

HLA and Ancestry: How It Works In Matching

Most cells in your body have distinct protein markers called HLA. These markers are used to match donors to patients in need of transplants. Compatible matches are most often found between donors and patients who have similar ancestry or geographic origins– which means an accurate description of both donor and patient ancestry is helpful to the matching process and saving lives.

How to Participate

If you choose to take part in this important study, please complete the online questionnaire by **clicking the orange button below**. This questionnaire will gather more detailed information on race and ancestry than you provided when you first joined the registry on [consent date]. When you click on the orange button below you will first be asked to consent to the study, and then you will be directed to the questionnaire.

Once you complete and submit the questionnaire, your responses will be compared with your genetic data that was typed when you joined the registry. This comparison will be used for scientific research, and will not affect your membership on the Be The Match Registry. If you choose not to participate in this study, you will still remain on the registry until the age of 61 unless you are unable or unwilling to donate.

YES, I WANT TO PARTICIPATE in the study

FREQUENTLY ASKED QUESTIONS about this study

If you have questions or would like more information about this research, please refer to the frequently asked questions by **clicking the blue button above**.

Thank you in advance for your participation in this study and continued commitment to Be The Match. This work will help us progress toward the goal of finding a match for every person in need of a transplant.

With gratitude,

Dennis L. Confer, M.D.
Chief Medical Officer

Pictured above are marrow and PBSC donors from left: Roymar, Erma and Bopper.

BeTheMatch.org | **Privacy Statement**
Unsubscribe | **Update your contact information**

If you unsubscribe, your email address will be removed from receiving the Be The Match Registry e-newsletter and other program updates. You will still be contacted by email, postal mail, telephone or other appropriate means if you become a potential match for a patient, or for administrative purposes, such as requesting that you update your registry contact information.

Be The Match® is operated by the National Marrow Donor Program®
3001 Broadway St. N.E., Suite 100, Minneapolis, MN 55413-1753 | 1 (800) MARROW-2

Table S2. Number of races (rows) and ancestries (columns) selected
(A) Considering all individuals. (B) Considering only populations with more than 50 individuals.

A.

	**1**	**2**	**3**	**4**	**5**	**6**	**7**	**8**	**9**	**10**	**11**	**12**	**13**	**14**	**15**	**16**	**Total**
**1**	40305	34549	13053	2810	524	102	38	12	1	3	-	1	-	-	-	-	91398
**2**	1224	4348	3211	1156	292	55	20	4	3	-	1	-	-	-	-	-	10314
**3**	88	206	542	345	132	32	15	5	2	1	-	-	-	-	-	-	1368
**4**	11	14	24	70	35	22	9	1	1	1	-	-	-	-	-	-	188
**5**	1	1	3	4	6	6	2	-	1	4	-	-	-	-	1	-	29
**6**	-	-	-	-	1	-	-	-	1	-	-	-	-	-	-	-	2
**7**	-	-	-	-	-	-	-	-	-	-	-	-	-	-	-	-	-
**Total**	41629	39118	16833	4385	990	217	84	22	9	9	1	1			1		103299

B.

	**1**	**2**	**3**	**4**	**5**	**6**	**7**	**8**	**9**	**10**	**11**	**12**	**13**	**14**	**15**	**16**	**Total**
**1**	39913	33144	11307	1918	61	-	-	-	-	-	-	-	-	-	-	-	86343
**2**	622	3103	1845	253	-	-	-	-	-	-	-	-	-	-	-	-	5823
**3**	-	-	107	-	-	-	-	-	-	-	-	-	-	-	-	-	107
**4**	-	-	-	-	-	-	-	-	-	-	-	-	-	-	-	-	-
**5**	-	-	-	-	-	-	-	-	-	-	-	-	-	-	-	-	-
**6**	-	-	-	-	-	-	-	-	-	-	-	-	-	-	-	-	-
**7**	-	-	-	-	-	-	-	-	-	-	-	-	-	-	-	-	-
**Total**	40535	36247	13259	2171	61	-	-	-	-	-	-	-	-	-	-	-	92273

Table S3. Correlation of fractional family ancestry (FFA) with personal ancestry salience (PAS).

Ancestry	Correlation	P-value
**South Asia**	0.94	< 0.001
**East Asia**	0.92	< 0.001
**Middle East**	0.89	< 0.001
**Southeast Asia**	0.83	< 0.001
**Eastern Europe**	0.80	< 0.001
**Northern Europe**	0.75	< 0.001
**Western Europe**	0.74	< 0.001
**Southern Europe**	0.73	< 0.001
**Caribbean**	0.73	< 0.001
**Central or South America**	0.72	< 0.001
**Scandinavia**	0.69	< 0.001
**Pacific Islands**	0.64	< 0.001
**Sub-Saharan Africa**	0.62	< 0.001
**African-American**	0.61	< 0.001
**American Indian**	0.41	< 0.001

Consent form

UNIVERSITY OF CALIFORNIA, SAN FRANCISCO
CONSENT TO BE IN RESEARCH

Study Title: Mapping the Intersection: Self-Identification and Genetic Ancestry

This is a research study, and you do not have to take part. You are being asked to take part in this study because you are registered with the National Marrow Donor Program (NMDP).

In this study, the researchers are doing a survey to learn more about the relationship between how people identify themselves with respect to race and ethnicity, and what their genetics tell us about their ancestry. The NMDP already has your genetic data for the genes involved in transplant, so there is no need to collect that information again. These data will be analyzed as part of this study.The National Human Genome Research Institute is paying for this research. About 50,000-100,000 people will participate in this study.

What will happen if I take part in this study?
If you agree to be in this study, you will complete a survey that begins on the next web page. The survey asks about your ancestry. It will take you about 5-10 minutes to complete the survey.

Are there any risks to me or my privacy?
Some of the survey questions may make you feel uncomfortable or raise unpleasant memories. You are free to skip any question.

We will do our best to protect the information we collect from you. Information that identifies you will be kept secure. The survey itself will not ask for details that directly identify you, such as your name or address. Please do not put this information on your survey. The completed surveys will be kept secure and separate from information that identifies you, and only a small number of researchers will have direct access to completed surveys. If this study is published or presented at scientific meetings, names and other information that might identify you will not be used.

Are there benefits?
There is no benefit to you. The survey results will be used for research.

Can I say "No"?
Yes, you do not have to complete the survey. If you choose not to be in this study you will remain registered as a potential bone marrow donor.

Are there any payments or costs?
You will not be paid for completing the survey. There are no costs to you.

Who can answer my questions about the study?
Answers to many questions may be found in the study frequently asked questions (FAQ): http://bethematch.org/HD/Ancestry-Study-FAQ/

You can talk with the study researcher about any questions not answered by the FAQ, concerns, or complaints you have about this study. Contact the study researcher Dr. Jill Hollenbach at 415-502-7289.

If you wish to ask questions about the study or your rights as a research participant to someone other than the researchers or if you wish to voice any problems or concerns you may have about the study, please call the Office of the Committee on Human Research at 415-476-1814.

CONSENT
PARTICIPATION IN RESEARCH IS VOLUNTARY.

You can print a copy of this consent form to keep for your records.

If you wish to be in this study, please click “Continue.”

- ☐ CONTINUE
- ☐ NO- I do not wish to participate in this study

Thank you for agreeing to participate in this important research study!

While taking the survey, *please do not use the back and forward buttons on your browser.* Once you have finished answering each question, click the button with the two arrows shown in the lower right hand corner of your screen to move on to the next question. You will not be able to return to previous questions during the survey, so please read each question carefully before you respond.

We appreciate your time and attention.

Race and Ethnicity

What is your race?

Mark <u>one or more</u> boxes to show the racial or ethnic group(s) you use to describe yourself.

- ☐ American Indian or Alaska Native
- ☐ Asian
- ☐ Black or African American
- ☐ Hispanic or Latino
- ☐ Native Hawaiian or other Pacific Islander
- ☐ White
- ☐ Other (please specify) ________

How do other people in this country typically classify you?

Mark one selection to show the racial or ethnic group most Americans would use to describe you.

- » American Indian or Alaska Native
- » Asian
- » Black or African American
- » Hispanic or Latino
- » Native Hawaiian or other Pacific Islander
- » White
- » Other (please specify)

Ancestry

Were you born in the United States?

- Yes
- No

Where were your parents and grandparents born?

Please include a response for each parent and grandparent.

	Born in the U.S.	Born outside the U.S.	Don't know
Father			
Mother			
Paternal grandfather (your father's father)			
Paternal grandmother (your father's mother)			
Maternal grandfather (your mother's father)			
Maternal grandmother (your mother's mother)			

From what countries or parts of the world did your ancestors come?

Select as many categories from the list below as needed to fully describe the origins of your family. If all of your grandparents were born in the United States, answer based on where your ancestors came from before they arrived in North America.

- ☐ Western Europe
  (England, Ireland, France, Germany, The Netherlands, etc.)
- ☐ Southern Europe
  (Italy, Spain, Turkey, etc.)
- ☐ Eastern Europe
  (Czech Republic, Poland, Russia, etc.)
- ☐ Scandinavia
  (Denmark, Norway, Sweden, etc.)
- ☐ East Asia
  (China, Japan, Korea, etc.)
- ☐ South Asia
  (India, Pakistan, Sri Lanka, etc.)
- ☐ Southeast Asia
  (Indonesia, Philippines, Vietnam, etc.)
- ☐ Pacific Islands
  (Hawaii, Guam, Samoa, etc.)
- ☐ Caribbean
  (Cuba, Puerto Rico, Trinidad and Tobago, etc.)
- ☐ Central or South America
  (Mexico, Nicaragua, Peru, etc.)
- ☐ Middle East
  (Iran, Lebanon, Saudi Arabia, etc.)
- ☐ Northern Africa
  (Egypt, Libya, Morocco, etc.)
- ☐ Sub-Saharan Africa
  (Kenya, Nigeria, Zimbabwe, etc.)
- ☐ American Indian
  (Navajo, Mayan, Tlingit, etc.)
- ☐ African American
- ☐ I do not know some, or all, of my family origins

Suppose you could describe a person using 100 points to represent all of their ancestries. For example, if you thought someone was mostly African but also part Latino, you might allocate 90 points for Sub-Saharan African and 10 points for Central or South American.

How would you describe your ancestry using this 100-point system?

Please indicate the number of points from 1-100 for each of the family origins shown below to represent its relative contributions to your ancestry.

Western Europe<br>(England, Ireland, France, Germany, The Netherlands, etc.)	0
Southern Europe<br>(Italy, Spain, Turkey, etc.)	0
Eastern Europe<br>(Czech Republic, Poland, Russia, etc.)	0
Scandinavia<br>(Denmark, Norway, Sweden, etc.)	0
East Asia<br>(China, Japan, Korea, etc.)	0
South Asia<br>(India, Pakistan, Sri Lanka, etc.)	0
Southeast Asia<br>(Indonesia, Philippines, Vietnam, etc.)	0
Pacific Islands<br>(Hawaii, Guam, Samoa, etc.)	0
Caribbean<br>(Cuba, Puerto Rico, Trinidad and Tobago, etc.)	0
Central or South America<br>(Mexico, Nicaragua, Peru, etc.)	0
Middle East<br>(Iran, Lebanon, Saudi Arabia, etc.)	0
Northern Africa<br>(Egypt, Libya, Morocco, etc.)	0
Sub-Saharan Africa<br>(Kenya, Nigeria, Zimbabwe, etc.)	0
American Indian<br>(Navajo, Mayan, Tlingit, etc.)	0
African American	0
Unknown	0
**Total**	0

Many Americans can trace their ancestry to several different parts of the world. To better understand your family history, we would like to ask about the ancestry of a few specific biological relatives.

Do you know the origin(s) or ancestry of one or more of your grandparents?

Please select all that apply from the list below.

- ☐ Yes, my paternal grandfather (biological father's biological father)
- ☐ Yes, my paternal grandmother (biological father's biological mother)
- ☐ Yes, my maternal grandfather (biological mother's biological father)

- [ ] Yes, my maternal grandmother (biological mother's biological mother)
- [ ] No, I do not know the ancestry of any of my biological grandparents

What ancestry or origin(s) best describe your paternal grandfather?

- [ ] Western Europe
  (England, Ireland, France, Germany, The Netherlands, etc.)
- [ ] Southern Europe
  (Italy, Spain, Turkey, etc.)
- [ ] Eastern Europe
  (Czech Republic, Poland, Russia, etc.)
- [ ] Scandinavia
  (Denmark, Norway, Sweden, etc.)
- [ ] East Asia
  (China, Japan, Korea, etc.)
- [ ] South Asia
  (India, Pakistan, Sri Lanka, etc.)
- [ ] Southeast Asia
  (Indonesia, Philippines, Vietnam, etc.)
- [ ] Pacific Islands
  (Hawaii, Guam, Samoa, etc.)
- [ ] Caribbean
  (Cuba, Puerto Rico, Trinidad and Tobago, etc.)
- [ ] Central or South America
  (Mexico, Nicaragua, Peru, etc.)
- [ ] Middle East
  (Iran, Lebanon, Saudi Arabia, etc.)
- [ ] Northern Africa
  (Egypt, Libya, Morocco, etc.)
- [ ] Sub-Saharan Africa
  (Kenya, Nigeria, Zimbabwe, etc.)
- [ ] American Indian
  (Navajo, Mayan, Tlingit, etc.)
- [ ] African American
- [ ] I do not know some of my paternal grandfather's origins

What ancestry or origin(s) best describe your paternal grandmother?

- [ ] Western Europe
  (England, Ireland, France, Germany, The Netherlands, etc.)

- Southern Europe
  (Italy, Spain, Turkey, etc.)
- Eastern Europe
  (Czech Republic, Poland, Russia, etc.)
- Scandinavia
  (Denmark, Norway, Sweden, etc.)
- East Asia
  (China, Japan, Korea, etc.)
- South Asia
  (India, Pakistan, Sri Lanka, etc.)
- Southeast Asia
  (Indonesia, Philippines, Vietnam, etc.)
- Pacific Islands
  (Hawaii, Guam, Samoa, etc.)
- Caribbean
  (Cuba, Puerto Rico, Trinidad and Tobago, etc.)
- Central or South America
  (Mexico, Nicaragua, Peru, etc.)
- Middle East
  (Iran, Lebanon, Saudi Arabia, etc.)
- Northern Africa
  (Egypt, Libya, Morocco, etc.)
- Sub-Saharan Africa
  (Kenya, Nigeria, Zimbabwe, etc.)
- American Indian
  (Navajo, Mayan, Tlingit, etc.)
- African American
- I do not know some of my paternal grandmother's origins

What ancestry or origin(s) best describe your maternal grandfather?

- Western Europe
  (England, Ireland, France, Germany, The Netherlands, etc.)
- Southern Europe
  (Italy, Spain, Turkey, etc.)
- Eastern Europe
  (Czech Republic, Poland, Russia, etc.)
- Scandinavia
  (Denmark, Norway, Sweden, etc.)
- East Asia
  (China, Japan, Korea, etc.)
- South Asia

(India, Pakistan, Sri Lanka, etc.)

- Southeast Asia
  (Indonesia, Philippines, Vietnam, etc.)
- Pacific Islands
  (Hawaii, Guam, Samoa, etc.)
- Caribbean
  (Cuba, Puerto Rico, Trinidad and Tobago, etc.)
- Central or South America
  (Mexico, Nicaragua, Peru, etc.)
- Middle East
  (Iran, Lebanon, Saudi Arabia, etc.)
- Northern Africa
  (Egypt, Libya, Morocco, etc.)
- Sub-Saharan Africa
  (Kenya, Nigeria, Zimbabwe, etc.)
- American Indian
  (Navajo, Mayan, Tlingit, etc.)
- African American
- I do not know some of my maternal grandfather's origins

What ancestry or origin(s) best describe your maternal grandmother?

- Western Europe
  (England, Ireland, France, Germany, The Netherlands, etc.)
- Southern Europe
  (Italy, Spain, Turkey, etc.)
- Eastern Europe
  (Czech Republic, Poland, Russia, etc.)
- Scandinavia
  (Denmark, Norway, Sweden, etc.)
- East Asia
  (China, Japan, Korea, etc.)
- South Asia
  (India, Pakistan, Sri Lanka, etc.)
- Southeast Asia
  (Indonesia, Philippines, Vietnam, etc.)
- Pacific Islands
  (Hawaii, Guam, Samoa, etc.)
- Caribbean
  (Cuba, Puerto Rico, Trinidad and Tobago, etc.)
- Central or South America
  (Mexico, Nicaragua, Peru, etc.)

- Middle East
  (Iran, Lebanon, Saudi Arabia, etc.)
- Northern Africa
  (Egypt, Libya, Morocco, etc.)
- Sub-Saharan Africa
  (Kenya, Nigeria, Zimbabwe, etc.)
- American Indian
  (Navajo, Mayan, Tlingit, etc.)
- African American
- I do not know some of my maternal grandmother's origins

Do you know the origin(s) or ancestry of either of your biological parents?

Please select all that apply from the list below.

- Yes, my biological mother
- Yes, my biological father
- I do not know the ancestry of either of my biological parents

What ancestry or origin(s) best describe your biological mother?

- Western Europe
  (England, Ireland, France, Germany, The Netherlands, etc.)
- Southern Europe
  (Italy, Spain, Turkey, etc.)
- Eastern Europe
  (Czech Republic, Poland, Russia, etc.)
- Scandinavia
  (Denmark, Norway, Sweden, etc.)
- East Asia
  (China, Japan, Korea, etc.)
- South Asia
  (India, Pakistan, Sri Lanka, etc.)
- Southeast Asia
  (Indonesia, Philippines, Vietnam, etc.)
- Pacific Islands
  (Hawaii, Guam, Samoa, etc.)
- Caribbean
  (Cuba, Puerto Rico, Trinidad and Tobago, etc.)
- Central or South America

(Mexico, Nicaragua, Peru, etc.)

- [ ] Middle East
  (Iran, Lebanon, Saudi Arabia, etc.)
- [ ] Northern Africa
  (Egypt, Libya, Morocco, etc.)
- [ ] Sub-Saharan Africa
  (Kenya, Nigeria, Zimbabwe, etc.)
- [ ] American Indian
  (Navajo, Mayan, Tlingit, etc.)
- [ ] African American
- [ ] I do not know some of my biological mother's origins

What ancestry or origin(s) best describe your biological father?

- [ ] Western Europe
  (England, Ireland, France, Germany, The Netherlands, etc.)
- [ ] Southern Europe
  (Italy, Spain, Turkey, etc.)
- [ ] Eastern Europe
  (Czech Republic, Poland, Russia, etc.)
- [ ] Scandinavia
  (Denmark, Norway, Sweden, etc.)
- [ ] East Asia
  (China, Japan, Korea, etc.)
- [ ] South Asia
  (India, Pakistan, Sri Lanka, etc.)
- [ ] Southeast Asia
  (Indonesia, Philippines, Vietnam, etc.)
- [ ] Pacific Islands
  (Hawaii, Guam, Samoa, etc.)
- [ ] Caribbean
  (Cuba, Puerto Rico, Trinidad and Tobago, etc.)
- [ ] Central or South America
  (Mexico, Nicaragua, Peru, etc.)
- [ ] Middle East
  (Iran, Lebanon, Saudi Arabia, etc.)
- [ ] Northern Africa
  (Egypt, Libya, Morocco, etc.)
- [ ] Sub-Saharan Africa
  (Kenya, Nigeria, Zimbabwe, etc.)
- [ ] American Indian
  (Navajo, Mayan, Tlingit, etc.)

- African American
- I do not know some of my biological father's origins

Knowledge check

How much would you say you know about your family history on your biological mother's side?

- Nothing at all
- A little
- A lot

How much would you say you know about your family history on your biological father's side?

- » Nothing at all
- » A little
- » A lot

Have you ever done any of the following to seek out information about your ancestry or family history?

Please select all that apply from the list below.

- Asked family members questions about family history
- Gone through family documents to find information
- Used a genealogy website, such as ancestry.com, familysearch.org
- Sent away for birth, death or marriage certificates or other official documents
- Gone to a library or archive to look for family records
- Taken a genetic ancestry test
- Other (please specify)
- None of these

Confidence family history

Some families do not talk about or are unaware of all their ancestral origins. On a scale from 1

(extremely unlikely) to 5 (extremely likely), *how likely do you think it is that you have any of the following ancestries:*

	Extremely unlikely	Very unlikely	Neither likely nor unlikely	Very likely	Extremely likely
African	○	○	○	○	○
American Indian	○	○	○	○	○
East Asian	○	○	○	○	○
South Asian	○	○	○	○	○
Jewish	○	○	○	○	○
Middle Eastern	○	○	○	○	○
Scandinavian	○	○	○	○	○
Southern European	○	○	○	○	○
Eastern European	○	○	○	○	○
Western European	○	○	○	○	○

Ancestry testing

How familiar are you with genetic ancestry testing? Would you say you are...

- ○ Very familiar
- ○ Somewhat familiar
- ○ Somewhat unfamiliar
- ○ Very unfamiliar

If you were offered a free genetic ancestry test, would you be interested in taking it?

- ○ Yes
- ○ No

Please share with us the reason(s) why you would not be interested in taking a genetic ancestry test.

If you took a genetic ancestry test and were told your ancestors were from one or more of these origins, how happy would you be?

For each ancestry, please indicate your level of happiness, from very unhappy to very happy.

	Very unhappy	Unhappy	Neither happy nor unhappy	Happy	Very happy
» African	○	○	○	○	○
» American Indian	○	○	○	○	○
» East Asian	○	○	○	○	○
» South Asian	○	○	○	○	○
» Jewish	○	○	○	○	○
» Middle Eastern	○	○	○	○	○
» Scandinavian	○	○	○	○	○
» Southern European	○	○	○	○	○
» Eastern European	○	○	○	○	○
» Western European	○	○	○	○	○

Basic Demographics

Around the time you were 16, were you living with both your biological mother and father?

- ○ Yes, both
- ○ No, biological mother only
- ○ No, biological father only
- ○ No, neither

What was the highest educational degree your mother received?

- ○ Did not finish high school

- High school degree or equivalent
- Associate's degree
- Bachelor's degree
- Graduate or professional degree
- Don't know

What was the highest educational degree your father received?

- » Did not finish high school
- » High school degree or equivalent
- » Associate's degree
- » Bachelor's degree
- » Graduate or professional degree
- » Don't know

What is the highest educational degree you have received?

- Did not finish high school
- High school degree or equivalent
- Associate's degree
- Bachelor's degree
- Graduate or professional degree

Please select the state where you lived around the time you were 16.

Please select the state where you currently live.

What is your age?

- 18 to 24 years
- 25 to 34 years
- 35 to 44 years
- 45 to 54 years
- 55 to 64 years
- Age 65 or older

Open-ended

Is there any information about your race or ancestry that you would like to share with us, that was not captured by the previous questions or answer options?

Please provide any additional details that you think would be important to fully understand your family origins and ancestry.

Supplementary References

1. *networkD3: D3 JavaScript Network Graphs from R* [computer program]. Version R package version 0.4. https://CRAN.R-project.org/package=networkD32017.
2. Wickham H. *Ggplot2 : elegant graphics for data analysis.* New York: Springer; 2009.
3. Spellman S, Setterholm M, Maiers M, et al. Advances in the selection of HLA-compatible donors: refinements in HLA typing and matching over the first 20 years of the National Marrow Donor Program Registry. *Biology of blood and marrow transplantation : journal of the American Society for Blood and Marrow Transplantation.* Sep 2008;14(9 Suppl):37-44.
4. Paunic V, Gragert L, Schneider J, Muller C, Maiers M. Charting improvements in US registry HLA typing ambiguity using a typing resolution score. *Human immunology.* Jul 2016;77(7):542-549.
5. Madbouly A, Gragert L, Freeman J, et al. Validation of statistical imputation of allele-level multilocus phased genotypes from ambiguous HLA assignments. *Tissue antigens.* Sep 2014;84(3):285-292.
6. Gragert L, Madbouly A, Freeman J, Maiers M. Six-locus high resolution HLA haplotype frequencies derived from mixed-resolution DNA typing for the entire US donor registry. *Human immunology.* Oct 2013;74(10):1313-1320.
7. Alexander DH, Novembre J, Lange K. Fast model-based estimation of ancestry in unrelated individuals. *Genome research.* Sep 2009;19(9):1655-1664.
8. Cann HM, de Toma C, Cazes L, et al. A human genome diversity cell line panel. *Science.* Apr 12 2002;296(5566):261-262.
9. Cavalli-Sforza LL. The Human Genome Diversity Project: past, present and future. *Nature reviews. Genetics.* Apr 2005;6(4):333-340.
10. Purcell S, Neale B, Todd-Brown K, et al. PLINK: a tool set for whole-genome association and population-based linkage analyses. *American journal of human genetics.* Sep 2007;81(3):559-575.
11. Ball CA, Barber MJ, Byrnes JK, et al. Ethnicity Estimate White Paper. ancestryDNA; 2013.
12. Venables WN, Ripley BD, Venables WN. *Modern applied statistics with S.* 4th ed. New York: Springer; 2002.
13. Jombart T. adegenet: a R package for the multivariate analysis of genetic markers. *Bioinformatics.* Jun 1 2008;24(11):1403-1405.
14. Jombart T, Ahmed I. adegenet 1.3-1: new tools for the analysis of genome-wide SNP data. *Bioinformatics.* Nov 1 2011;27(21):3070-3071.
