# Supplementary material for: Multiple measures for self-identification improve matching donors with patients in unrelated hematopoietic stem cell transplant
Source: Commun Med (Lond). 2024 Oct 3;4:189. doi: 10.1038/s43856-024-00620-w (PMC11449941; doi:10.1038/s43856-024-00620-w)
Supplement: Supplementary file 2 — Description of Additional Supplementary Files [file 43856_2024_620_MOESM2_ESM.pdf]

Description of Additional Supplementary Files

File name: Supplementary Data Table 1

File description: Processed data underlying all figures and tables**.**
