# Supplementary material for: Multiple measures for self-identification improve matching donors with patients in unrelated hematopoietic stem cell transplant
Source: Commun Med (Lond). 2024 Oct 3;4:189. doi: 10.1038/s43856-024-00620-w (PMC11449941; doi:10.1038/s43856-024-00620-w)
Supplement: Supplementary file 4 — Reporting Summary [file 43856_2024_620_MOESM4_ESM.pdf]

nature portfolio

Corresponding author(s): Jill A. Hollenbach

Last updated by author(s): Jun 19, 2024

Reporting Summary

Nature Portfolio wishes to improve the reproducibility of the work that we publish. This form provides structure for consistency and transparency in reporting. For further information on Nature Portfolio policies, see our Editorial Policies and the Editorial Policy Checklist.

Statistics

For all statistical analyses, confirm that the following items are present in the figure legend, table legend, main text, or Methods section.

n/a	Confirmed	
☐	☒	The exact sample size (*n*) for each experimental group/condition, given as a discrete number and unit of measurement
☐	☒	A statement on whether measurements were taken from distinct samples or whether the same sample was measured repeatedly
☐	☒	The statistical test(s) used AND whether they are one- or two-sided<br>*Only common tests should be described solely by name; describe more complex techniques in the Methods section.*
☐	☒	A description of all covariates tested
☐	☒	A description of any assumptions or corrections, such as tests of normality and adjustment for multiple comparisons
☐	☒	A full description of the statistical parameters including central tendency (e.g. means) or other basic estimates (e.g. regression coefficient) AND variation (e.g. standard deviation) or associated estimates of uncertainty (e.g. confidence intervals)
☐	☒	For null hypothesis testing, the test statistic (e.g. *F*, *t*, *r*) with confidence intervals, effect sizes, degrees of freedom and *P* value noted<br>*Give P values as exact values whenever suitable.*
☐	☒	For Bayesian analysis, information on the choice of priors and Markov chain Monte Carlo settings
☒	☐	For hierarchical and complex designs, identification of the appropriate level for tests and full reporting of outcomes
☒	☐	Estimates of effect sizes (e.g. Cohen's *d*, Pearson's *r*), indicating how they were calculated

Our web collection on statistics for biologists contains articles on many of the points above.

Software and code

Policy information about availability of computer code

Data collection	All survey data were collected in Qualtrics
Data analysis	All data analysis was conducted in R, code available at https://github.com/Hollenbach-lab/AQP_Paper1_PublicRelease

For manuscripts utilizing custom algorithms or software that are central to the research but not yet described in published literature, software must be made available to editors and reviewers. We strongly encourage code deposition in a community repository (e.g. GitHub). See the Nature Portfolio guidelines for submitting code & software for further information.

Data

Policy information about availability of data

All manuscripts must include a data availability statement. This statement should provide the following information, where applicable:

- Accession codes, unique identifiers, or web links for publicly available datasets
- A description of any restrictions on data availability
- For clinical datasets or third party data, please ensure that the statement adheres to our policy

The data that support the findings of this study are not openly available due to reasons of sensitivity and are available from the corresponding author upon reasonable request. Data are located in controlled access data storage at the University of California San Francisco. Processed data underlying tables and figures is provided in Supplementary Data Table 1.

Human research participants

Policy information about studies involving human research participants and Sex and Gender in Research.

Reporting on sex and gender	Data for gender was provided by participants at time of enrolling in the donor registry, and is provided in demographics given in Tables 1 and 2.
Population characteristics	Demographic data is provided in Tables 1 and 2.
Recruitment	We collected multiple self-reported measures of race and ancestry from a cohort of more than 100,000 U.S. adults who also provided genetic data for HLA as potential donors registered with the National Marrow Donor Program (NMDP). To ascertain genetic ancestry, we used the registry's data for the human leukocyte antigen (HLA) complex on the short arm of chromosome 6, which is critical to matching in tissue transplant. The HLA loci exhibit extreme levels of variability and differentiation among human populations and the region is relatively well-maintained during gametogenesis, and thus can be used as ancestry informative markers19-22. Our survey of potential NMDP donors, conducted for this study in spring 2015, included questions about racial self-identification and multiple (geographic) ancestry items.
Ethics oversight	All participants provided informed consent (available in Supplementary files) and this study was approved by the Institutional Review Board at the University of California San Francisco (study #14-13977).<br><br>All participants provided informed consent (available in Supplementary files) and this study was approved by the Institutional Review Board at the University of California San Francisco (study #14-13977).

Note that full information on the approval of the study protocol must also be provided in the manuscript.

Field-specific reporting

Please select the one below that is the best fit for your research. If you are not sure, read the appropriate sections before making your selection.

☒ Life sciences ☐ Behavioural & social sciences ☐ Ecological, evolutionary & environmental sciences

For a reference copy of the document with all sections, see nature.com/documents/nr-reporting-summary-flat.pdf

Life sciences study design

All studies must disclose on these points even when the disclosure is negative.

Sample size	Sample size was based on survey participation.
Data exclusions	All participants who completed the survey were included.
Replication	NA
Randomization	NA
Blinding	NA

Reporting for specific materials, systems and methods

We require information from authors about some types of materials, experimental systems and methods used in many studies. Here, indicate whether each material, system or method listed is relevant to your study. If you are not sure if a list item applies to your research, read the appropriate section before selecting a response.

Materials & experimental systems

n/a	Involved in the study
☒	☐ Antibodies
☒	☐ Eukaryotic cell lines
☒	☐ Palaeontology and archaeology
☒	☐ Animals and other organisms
☒	☐ Clinical data
☒	☐ Dual use research of concern

Methods

n/a	Involved in the study
☒	☐ ChIP-seq
☒	☐ Flow cytometry
☒	☐ MRI-based neuroimaging
